# A Glycosaminoglycan Based, Modular Tissue Scaffold System for Rapid Assembly of Perfusable, High Cell Density, Engineered Tissues

**DOI:** 10.1371/journal.pone.0084287

**Published:** 2014-01-20

**Authors:** Ramkumar Tiruvannamalai-Annamalai, David Randall Armant, Howard W. T. Matthew

**Affiliations:** 1 Department of Biomedical Engineering, Wayne State University, Detroit, Michigan, United States of America; 2 Departments of Obstetrics & Gynecology, Wayne State University, Detroit, Michigan, United States of America; 3 Program in Reproductive & Adult Endocrinology, National Institute of Child Health & Human Development, National Institutes of Health, Bethesda, Maryland, United States of America; 4 Department of Chemical Engineering and Materials Science, Wayne State University, Detroit, Michigan, United States of America; Michigan State University, United States of America

## Abstract

The limited ability to vascularize and perfuse thick, cell-laden tissue constructs has hindered efforts to engineer complex tissues and organs, including liver, heart and kidney. The emerging field of modular tissue engineering aims to address this limitation by fabricating constructs from the bottom up, with the objective of recreating native tissue architecture and promoting extensive vascularization. In this paper, we report the elements of a simple yet efficient method for fabricating vascularized tissue constructs by fusing biodegradable microcapsules with tunable interior environments. Parenchymal cells of various types, (i.e. trophoblasts, vascular smooth muscle cells, hepatocytes) were suspended in glycosaminoglycan (GAG) solutions (4%/1.5% chondroitin sulfate/carboxymethyl cellulose, or 1.5 wt% hyaluronan) and encapsulated by forming chitosan-GAG polyelectrolyte complex membranes around droplets of the cell suspension. The interior capsule environment could be further tuned by blending collagen with or suspending microcarriers in the GAG solution These capsule modules were seeded externally with vascular endothelial cells (VEC), and subsequently fused into tissue constructs possessing VEC-lined, inter-capsule channels. The microcapsules supported high density growth achieving clinically significant cell densities. Fusion of the endothelialized, capsules generated three dimensional constructs with an embedded network of interconnected channels that enabled long-term perfusion culture of the construct. A prototype, engineered liver tissue, formed by fusion of hepatocyte-containing capsules exhibited urea synthesis rates and albumin synthesis rates comparable to standard collagen sandwich hepatocyte cultures. The capsule based, modular approach described here has the potential to allow rapid assembly of tissue constructs with clinically significant cell densities, uniform cell distribution, and endothelialized, perfusable channels.

## Introduction

Fabrication of 3D constructs that promote cell-cell interaction, extra cellular matrix (ECM) deposition and tissue level organization is a primary goal of tissue engineering [1]. Accomplishing these prerequisites with the currently available conventional scaffolds and fabrication techniques still remains a challenge. Some of the tissue types that have been successfully engineered include skin [2], bone [3]–[5] and cartilage [4], [6], [7]. Significant success has also been achieved in nerve regeneration [8], corneal construction [9]–[11] and vascular tissue engineering [12]; However, the success rate has been relatively low in engineering complex tissue types such as liver, lung, and kidney due to their complex architectures and metabolic activities.

In conventional preformed scaffolds, the cell viability depends on diffusion of oxygen, nutrients and growth factors from the surrounding host tissues, and it is limited to 100–200 microns thickness at cell densities comparable to that of normal tissues [13]. Hence in constructs with larger dimensions, efficient mass transfer and subsequent cell survival can be achieved only by significantly reducing cell densities or by tolerating hypoxic conditions. Moreover, in a porous scaffold, uniform distribution throughout the construct is difficult to achieve, and the seeded cells will stay on the peripheral surface of the construct forming a thin peripheral layer. In addition, these scaffolds cannot facilitate incorporation of multiple cell types in a controlled manner. Hence the slow vascularization, mass transfer limitation, low cell density and non-uniform cell distribution limits conventional methods from engineering large and more complex organs. Therefore, an innate structure that supports functional vascularization is imperative for engineering large tissues grafts. Many strategies have been proposed to incorporate vascular structure that includes creating endothelial microchannels inside scaffolds [14], [15], surface modification and/or controlled releasing of pro-vasculogenic growth factor and cytokines [16]–[18], coculturing vascular cell types for microvessel formation [19] etc. Despite their limited success, none of these approaches is able to incorporate an extensive vasculature as seen in natural organs.

**Figure 1:**
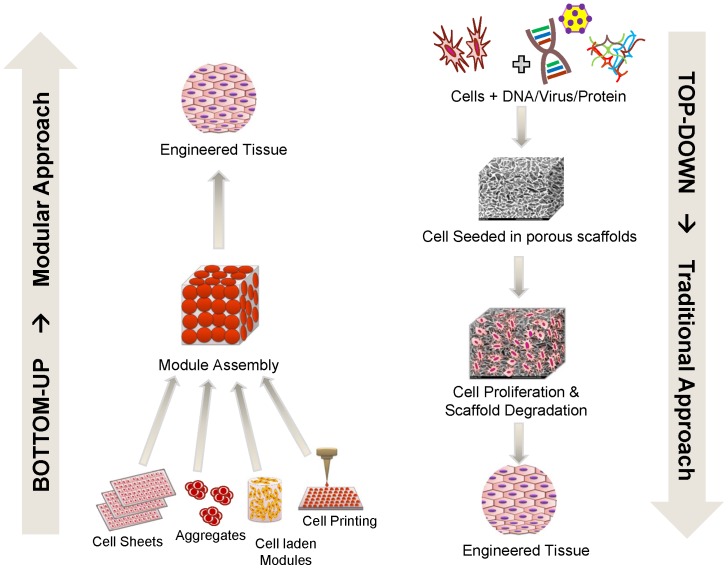
Bottom-up vs. top-down approaches in tissue engineering. The traditional, top-down approach (right) involves seeding cells into full sized porous scaffolds to form tissue constructs. This approach poses many limitations such as slow vascularization, diffusion limitations, low cell density and non-uniform cell distribution. In contrast, the modular or bottom-up approach (left) involves assembling small, non-diffusion limited, cell-laden modules to form larger structures and has the potential to eliminate the shortcomings of the traditional approach.

The bioinspired modular tissue engineering approach has emerged in recent years as a promising fabrication strategy to address the common shortcomings of a preformed scaffold by assembling tissue constructs from the bottom up [20], [21]. Using this principle, complex tissues and organs can be engineered efficiently from microscale modules as opposed to the top down approach of conventional scaffolds [21]. This approach is increasingly becoming a promising tool to study and recreate vascular physiology in tissue engineering applications [22], [23]. Some of the proposed modular TE strategies include 3D tissue printing [24]–[26], cell sheets technology [27] and assembly of cell laden hydrogels [20], [28] (Figure 1).

Here we report the development of a simple yet efficient method for assembling tissue prototypes with embedded, endothelialized channels by fusing microscale capsules. Our method of cell encapsulation was previously developed for perfusion culture of highly metabolic cells [29]–[33].We are now extending its use to fabricate modular tissue constructs. Capsules were seeded internally with various test cell types and externally with vascular endothelial cells to authenticate our proof of principle and growth and metabolic performance were studied. To enhance the versatility of these microcapsules, the effects of tuning the capsule interiors with collagen gels or microcarriers were explored.

## Materials and Methods

### Cell culture conditions

All chemical and culture reagents were purchased from Sigma-Aldrich unless mentioned otherwise. The human trophoblast cell line HTR-8/SVneo[34] (HTBs) was used as the model cell type for some studies due to their high proliferative capacity and ability to form dense, tissue-like aggregates. The cells were cultured in 10 cm tissue culture dishes, using F12/DMEM supplemented with 5% fetal bovine serum (FBS), 50 mg/ml gentamycin and 2.5 mg/L Amphotericin-B.

For co-culture studies, vascular smooth muscle cells (SMCs) were isolated from rat aorta and endothelial cells (AECs) were isolated from sheep aorta using established enzymatic procedures [2], [35], [36]. Sheep aortas were procured from a slaughterhouse under an educational license (Wolverine Packing Company, Detroit, MI). Aortas were obtained within 2 hours of slaughter and used for AEC isolation immediately. Human umbilical vein endothelial cells (HUVECs) obtained from ATCC (Manassas, VA) were also used as vascular component. Primary cells were used from passages 3 to 6. SMCs and AECs were maintained in DMEM supplemented with 10% FBS, 50 mg/ml gentamycin, and 2.5 mg/L Amphotericin-B. In addition, SMC cultures were supplemented with 2 ng/ml fibroblast growth factor 2 and AECs with 50 ng/ml of epidermal growth factor. For HUVECs, MCDB 131 medium supplemented with Endothelial Cell Growth Kit-VEGF (ATCC) was used. During co-cultures of parenchymal and vascular components, a 50–50 mixture of the respective culture media was used. Primary hepatocytes were isolated from Sprague dawley rats weighing 250–450 g by the two-step collagenase perfusion technique described by Seglen [37] and modified by Dunn [38], [39]. Cell viability averaged 90–95%, as assessed by trypan blue exclusion, and the average yield was 4×10^8^ viable cells per liver. Type I collagen was isolated from Sprague dawley rat tail tendons as previous described [38] and used for hepatocyte collagen sandwich cultures. Hepatocyte culture medium consisted of high glucose DMEM medium supplemented with 10% fetal bovine serum (FBS), 0.5 U/mL insulin, 7 ng/mL glucagon, 20 ng/mL epidermal growth factor, 7.5 µg/mL hydrocortisone, 100 mg/L gentamycin and 2.5 mg/L amphotericin B. Culture medium was collected and analyzed for albumin and urea synthesis using established methods [33], [40]. All dish cell cultures were maintained at 37°C in a 5% CO2/95% air humidified incubator.

### Ethics statement

Harvesting of rat hepatocytes and aortic smooth muscle cells for culture was carried out in strict accordance with the recommendations in the Guide for the Care and Use of Laboratory Animals of the National Institutes of Health. The cell isolation protocol was approved by the Animal Investigation Committee of Wayne State University (Protocol Number: A-07-16-10). Surgery and liver perfusion were performed under ketamine/xylazine anesthesia, and all efforts were made to minimize suffering.

### Biopolymer materials

The materials used in preparing our microcapsules and modular scaffolds were: chitosan from crab shells, molecular weight ∼600 kDa (Sigma); chondroitin 4-sulfate sodium salt from bovine trachea, molecular weight ∼50–100 kDa (Sigma); hyaluronic acid sodium salt from *Streptococcus equii*, molecular weight 1500–1800 kDa (Sigma); dextran sulfate sodium salt, molecular weight ∼500 kDa (SCBT); heparin sodium salt from porcine intestinal mucosa, molecular weight 17–19 kDa (Celsus); carboxymethylcellulose sodium salt, molecular weight 250 kDa (Sigma); polygalacturonic acid sodium salt (Sigma) and collagen type-I isolated from Sprague Dawley rat tail tendons (Invitrogen).

Aqueous solutions of the polyanions (chondroitin 4-sulfate (CSA), carboxymethylcellulose (CMC), hyaluronic acid (HA), polygalacturonic acid (PGA)) were prepared in a HEPES-sorbitol buffer containing: 0.4 g/L KC1, 0.5 g/L NaC1, 3.0 g/L HEPES-sodium salt, and 36 g/L sorbitol, pH 7.3. Polyanion solutions were sterilized by autoclaving at 121°C. The two formulations of polyanionic solutions studied for capsule formation were: (a) 4 wt% CSA/1.5 wt% CMC and (b) 1.0 or 1.5 wt% HA. To prepare the polycationic solution, chitosan powder was suspended in water (3 g in 250 ml) and autoclaved at 121°C. Under sterile conditions, 0.6 ml of glacial acetic acid was added to the aqueous suspension and stirred for 4 hours to partially dissolve the chitosan. Likewise, 19 g of sorbitol was autoclaved in 250 ml of water and then mixed with the chitosan solution. Undissolved chitosan was removed by centrifugation at 500 G. PGA (0.1 wt%) in HEPES-sorbitol buffer was used for surface stabilization of capsules. For capsule experiments employing collagen, cold collagen-I solution was diluted to 2 mg/ml in 1 mM HCl, and then neutralized with 10X DMEM (9∶1 ratio). Normal saline (0.9 wt% NaCl) was used for capsule washing immediately after formation.

### Cell encapsulation

**Figure 2:**
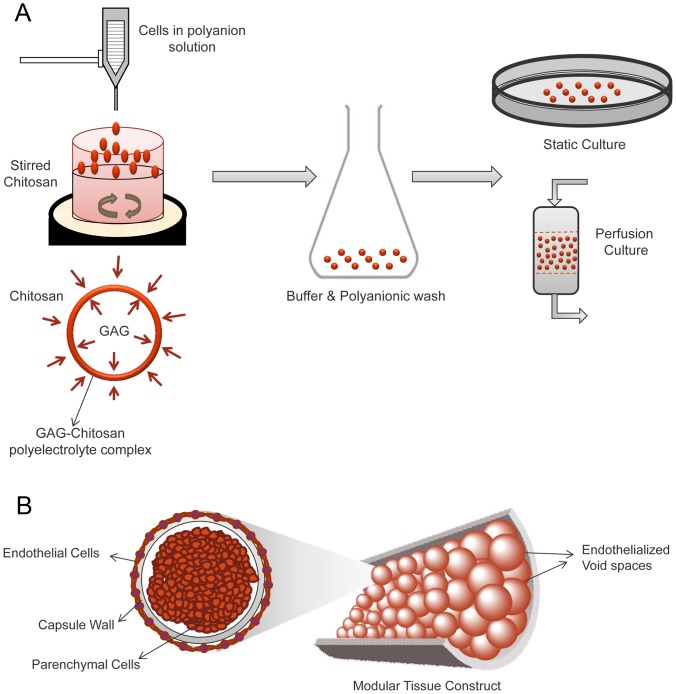
Microencapsulation through complex coacervation and modular assembly. (A) Droplets of cells suspended in a polyanionic solution were dispensed into a stirred chitosan solution. Ionic interactions between the oppositely charged polymers formed an insoluble ionic complex membrane at the droplet-solution interface, thus encapsulating the suspended cells. Capsule were washed surface-stabilized with a suitable anionic polymer solution, and transferred to culture. (B) Cell laden capsules can be assembled in a packed bed fashion with interconnected endothelialized channels that may enable perfusion of fluids such as blood with limited adverse reactions.

Cells were encapsulated in microcapsules produced by polyelectrolyte complexation between cationic chitosan and polyanions as described in detail previously [29], [30]. In brief, the 5–10 million cells were suspended in 1 ml of a polyanionic solution (either 4 wt% CSA/1.5 wt% CMC, or 1.5 wt% HA). Droplets of the cell suspension (∼0.8 mm diameter) were dispensed into 30 ml of stirred chitosan solution containing 2–3 drops of Tween 20. A 24 gauge Teflon catheter was used to generate droplets and filtered air was blown coaxially to shear away the droplets at a suitable size. Care was taken during encapsulation process to ensure uniform droplet size. Capsule membranes were formed almost instantaneously by ionic complexation between the oppositely charged polymers. Capsules were allowed to mature for ∼1 min in the stirred chitosan, followed by two washes with normal saline and surface stabilization by washing with 0.1% PGA solution. Microcapsules were subsequently equilibrated with culture medium for ∼60 min and then transferred to suitable culture conditions (Figure 2A).

The interior environment of the capsules could be enhanced with collagen gel or adhesion surface-providing microcarriers when desired. For capsules with an internal collagen matrix, chilled Type I collagen solution (1 mg/ml in 1 mM HCL) was neutralized with 10X DMEM in a 9∶1 ratio, and mixed with an equal volume of double strength polyanionic solution (e.g. 8% CSA/3% CMC). Cells were then suspended in this mixture instead of the regular polyanion solution, and capsules were made as described previously. For microcarrier co-encapsulation, PBS swelled microcarriers were suspended along with cells in normal strength polyanionic solution at a volume ratio of 0.5∶1 (packed cells+microcarriers:polyanion solution). The suspension was then dispensed as droplets to generate capsules as described above. Capsules enhanced with interior collagen or microcarriers were subjected to similar washing and surface stabilization steps as described above prior to culture.

### Endothelial cell seeding on capsule surfaces

Capsules were coated with an adsorbed layer of Type I collagen prior to externally seeding endothelial cells. For coating collagen on the outer surface, non-surface stabilized capsules (i.e. capsules without a PGA final wash) were washed in dilute acidic collagen solution (0.2 mg/ml of collagen in 1 mM acetic acid) for 1–2 min and then equilibrated with culture medium for 30 mins. Capsules that had been previously surface stabilized with PGA, were first washed with dilute chitosan solution (0.06% chitosan) prior to the dilute collagen wash. The equilibration culture medium was then removed and an endothelial cell suspension (HUVECs or AECs) in medium was added to settled capsules in a 50 ml centrifuge tube. Cells were seeded at a density of 10^6^ cells per ml of capsules and incubated at 37°C for 60 minutes with gentle resuspension every 10 min. After incubation, the seeded capsules were transferred to bioreactor chambers or tissue culture dishes for further experiments.

### Evaluation of capsule wall permeability

The permeability of capsule walls was studied fluorometrically by measuring the rate of diffusion of tetramethylrhodamine-labelled bovine serum albumin (BSA-TMR) from the capsules, as detailed before [29], [30], [41]. The rate was used to calculate an overall mass transfer coefficient for the capsule wall membrane under mixing conditions.

A precise number of capsules (100–150 per sample, n = 3) of similar size from each formulation were counted out and equilibrated in HBSS (pH 7.4) containing 2.5 mg/ml BSA (13% TMR-labeled. The equilibration saturated all the BSA binding sites of the capsule wall and efficiently loaded BSA into the capsules. After washing and resuspension in fresh HBSS, the capsules were redistributed into three fluorescence cuvettes (4 ml volume, 1 cm light path) at 35–50 capsules per cuvette. The HBSS volume was made up to 3 ml and the cuvettes were sealed and mixed horizontally on a linear shaker at 100 rpm. The outward diffusion of BSA was followed by measuring the fluorescence of the external HBSS at exitation/emission wavelengths of 541/572 nm at regular time intervals for 3 h. The BSA concentration was determined using a standard curve covering the range of 0–50 µg/mL total albumin. The overall mass-transfer coefficient for diffusion across the capsule wall (K) was calculated by solving the differential equation obtained through an unsteady state mass balance on the external solution [29]. 
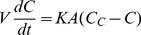
(1)

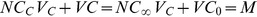
(2)


Where: K is the overall mass transfer coefficient for membrane diffusion; M is the total mass of solute present in the cuvette (M  =  C_r_ (V+NV_c_)); V and V_c_ are the volume of external solution and volume of capsules, respectively; N is the number of capsules; A is the total surface area (A  =  N*surface area of single capsule); C is the concentration of solute in external solution; C_o_ is the initial extracapsular concentration; C_c_ is the concentration of solute in the capsules; C_∞_ is the initial intracapsule concentration; C_r_ is the final concentration after end of 3 hours; and t is time. The solution to the equations 1 and 2 yields,
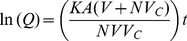
(3)


Where Q is a dimensionless concentration-dependent parameter defined as
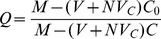



The overall mass-transfer coefficient for transmembrane diffusion, K, was determined by plotting ln(Q) vs. time and determining the slope of the linear portion of the curve by linear regression. The intrinsic permeability, P, of each capsule wall was determined from the relation:
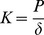



Where δ is the thickness of the capsule wall.

### Assembly of modular constructs

Individual capsules were fused into 3D constructs in one of two ways. In the first method, freshly formed capsules were fused by allowing them to sit in contact with each other after the second saline wash, but before the surface stabilizing PGA wash. Freshly formed capsules were washed once with normal saline and then transferred in saline to a cylindrical mold with a 250 micron mesh at the base. Capsules were allowed to settle within the mold and held stationary for 2–3 minutes to allow inter-capsule adhesion. The excess saline was then drained and the capsule surfaces in the fused construct were then stabilized by briefly rinsing with saline, followed by a diluted polyanion solution (i.e. 0.1% PGA or 0.4% CSA/0.15% CMC), followed by a final PBS rinse.

In the second fusion method, previously stabilized and cultured capsules were first reloaded with a polyanion by incubation in a diluted polyanion solution (0.1% heparin or 0.4% CSA/0.15% CMC). The capsules were then transferred to a cylindrical mold with the mesh base. After draining excess polyanion solution, the mold with reloaded capsules was perfused with 0.06 wt% chitosan solution to ionically fuse the capsules. Excess chitosan solution was drained, the capsules were rinsed with normal saline and surface stabilized by a brief perfusion with a dilute polyanion solution. The fused modular construct was then removed from the mold for further culture or analysis.

### Evaluation of cell proliferation inside capsules

Cell proliferation inside capsules was characterized using either a Hoechst DNA quantification assay [42] or an MTT assay. Briefly, 30 capsules were distributed into each well of a 24 well plate. Capsules were maintained under standard culture conditions, and one well was sacrificed at each time point. The capsules were gently ruptured using a fire-polished Pasteur pipette, and the cell aggregates within were lysed using cell lysis buffer (0.1% SDS, 10 mM Tris-HCl, 1 mM EDTA) to extract whole DNA. To an aliquot of this extract was added an equal volume of Hoechst 33258 dye dissolved at 1 mg/ml in TNE buffer (50 mM Tris-HCL, 100 mM NaCl, 0.1 mM EDTA). Fluorescence of the mixture was then measured (EX/EM 350/450 nm). A calf thymus DNA standard curve was used to determine the total DNA concentration. For the MTT proliferation assay, capsules were washed in PBS and suspended in phenol red free DMEM containing 2 mg/ml MTT. After incubation for 4 hours at 37°C, the solution was aspirated and 150 µL of DMSO was added to extract the formazan crystals. After 10 mins of rotary agitation, the absorbance of the DMSO extract was measured at 540 nm using a spectrophotometer. Exponential cell growth was assumed and the specific growth rate was determined by fitting the following equation to the absorbance reading: 
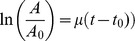



Where A_0_ and A are initial and final absorbance or fluorescence readings respectively, t_0_ and t are initial and final time points, and μ is the specific growth rate in time^−1^.

### Cell viability imaging and histology

Cell viability was assessed using Calcein-AM and ethidium homodimer (Cytotoxicity Kit L3224, Invitrogen). The cell laden capsules were washed with PBS and incubated in serum free DMEM containing 4 µM Calcein-AM and 4 µM ethidium homodimer for 20 min at 37°C. For long-term tracking of HUVECs on capsules and fused capsule constructs, CellTracker™ Green CMFDA (Invitrogen) was used. Briefly, adherent cells were rinsed with PBS and incubated in a serum free culture medium containing 5 µM CellTracker Green probe for 45–60 min. After the incubation the medium was replaced with pre-warmed normal medium and incubated for another 30 min for the dye to undergo modification due to intracellular esterases. The cells were then trypsinized and seeded onto capsule outer surface. Cell fluorescence was then observed using wide-field fluorescence microscopy and laser scanning confocal microscopy (Zeiss LSM-410).

The distribution and organization of cells and matrix inside the encapsulated cultures were investigated by histology. Cell laden individual capsules and fused capsule constructs were washed in PBS, fixed in 10% buffered formalin, dehydrated in an ethanol series, paraffin embedded, sectioned (4–6 µm) and stained using Hematoxylin and Eosin (H&E) or Masson's trichrome stains (Sigma-Aldrich). The stained sections were observed using bright field microscopy.

### Perfusion culture of encapsulated hepatocytes

Encapsulated primary rat hepatocytes (encapsulation density: 20×10^6^ cells/mL of CSA/CMC) were maintained in perfusion cultures under both packed bed and fluidized bed conditions as previously described [29], [33]. For fluidized perfusion, non-fused capsules were fluidized by a continuous upward flow of the culture medium in a cylindrical chamber within a continuous circulation flow circuit. For the packed bed cultures, the capsules were fused in a cylindrical flow chamber as described above and subjected to a downward flow of the medium in a continuous circulation flow circuit. Medium exiting the culture chamber was oxygenated using a silicone tubing oxygenator (supplied with 95% air/5% CO_2_) and recirculated using a peristaltic pump. The flow rates were adjusted to maintain physiological pressure differences (<100 mmHg) across the chamber (4–5 mL per minute). The perfusion system was maintained at 37°C for 1–2 weeks and medium was changed every 2–3 days. Medium samples were collected daily for evaluation of urea and albumin synthesis by the hepatocytes.

### Analysis of albumin and urea synthesis

Standard methods for measuring albumin and urea production rates were used to assess hepatocyte function. Culture medium collected from collagen sandwich cultures and perfusion bioreactor cultures at regular intervals was analyzed for rat serum albumin by ELISA with purified rat albumin (Sigma) and a peroxidase conjugated anti-rat albumin antibody (Bethyl). Urea production was quantified using the diacetylmonoxime method as previously described [43]. Standard curves for both quantification techniques were generated using purified rat albumin or urea dissolved in culture medium. Absorbances were measured with a Spectramax microplate reader.

### Statistical analyses

Measurements were performed in triplicate (n = 3). Data are plotted as means with error bars representing standard deviation. Statistical comparisons were done using Student's t-test with a 95% confidence limit. Differences with p<0.05 were considered statistically significant.

## Results

### High density encapsulated cell cultures

**Figure 3:**
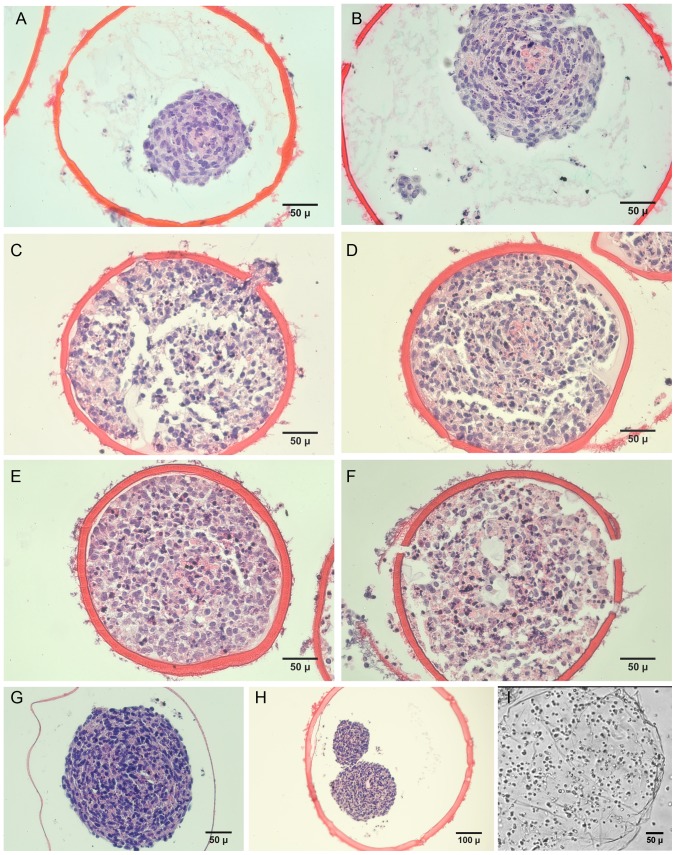
Histology of microencapsulated cultures of human trophoblasts (HTBs) in various GAG-chitosan capsule formulations. HTBs in CSA/CMC capsules on days (A) 5, (B) 10, (C) 15, (D) 20, (E) 25, (F) 30. (G) Hyaluronan/CMC capsules. (H) HA capsules. (I) Dextran sulfate/CMC capsules quickly ruptured due to osmotic swelling.

We investigated the effects of different polyanions and polyanion blends on the capsule characteristics and encapsulated cell growth patterns. The 4%CSA/1.5%CMC capsules (Figure 3 A–F) and the 1.5% HA capsules (Figure 3H) were sturdy and could be easily handled with forceps. In contrast, the 1.5%HA/1.5%CMC mixture formed very thin walled capsules (Figure 3G), many of which ruptured after a week of culture. Capsules made with 4% DXS or 3% CMC were thin walled and ruptured within 2–3 hours due to osmotic swelling (Figure 3I).

Encapsulated HTBs grew rapidly, eventually filling the capsules, and most cells appeared viable with a distinct nucleus up to at least day 30 (Figure 3 A, B, C, D, E, F). This indicated that the capsule wall was sufficiently permeable to nutrients to allow maintenance of a dense, tissue-like cell mass. The estimated capsule cell density (∼6×10^7^ cells/cm^3^, assessed via image analysis) at day-30 was high enough to replicate the cell density in many tissues. By the end of week-3, HTBs had invaded the capsule wall as seen in Figure 3C. This in vitro invasion suggests that the capsule materials may be degraded within a relatively short time frame upon implantation in vivo. No necrotic core was observed within the encapsulated cell mass at least until 45 days of static culture.

### Endothelial cell growth on capsule surfaces

**Figure 4:**
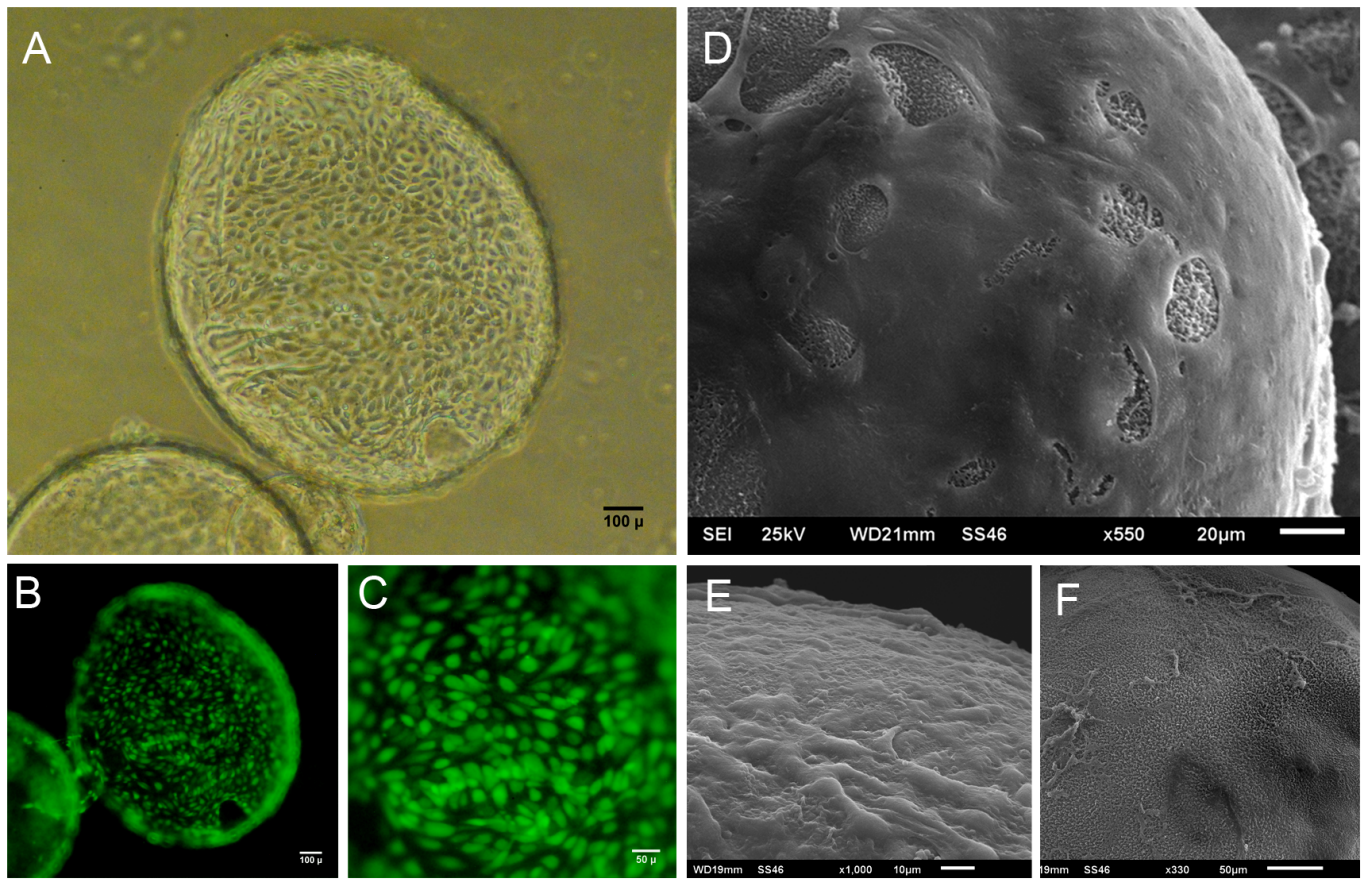
HUVECs seeded on CSA/CMC capsules after surface coating with collagen. (A) Phase contrast image of a capsule coated with a monolayer of HUVECs, 24 hours after cell seeding. (B,C) CellTracker Green fluorescence images of HUVECs seeded on the outer surface shown in A. (D,E) SEM images of HUVEC seeded capsule surfaces after 1 hour (E) and 24 hours (D). (F) Non-seeded capsule surface.

The growth of sheep aortic endothelial cells (AEC) and HUVECs was investigated by seeding these cells onto the outside surfaces of CSA/CMC capsules. Endothelial cells attached poorly to surface stabilized capsule surfaces. However, their attachment and growth greatly improved when type-I collagen was coated onto the outer surface of the CSA/CMC capsules. HUVECs seeded on the collagen coated CSA/CMC capsules attached well and formed a viable monolayer within 24 hours of seeding (Figure 4A, B, C). SEM images of capsules fixed 1 hour post-seeding (Figure 4E) showed a continuous, but irregular monolayer of cells in varying stages of spreading. SEM images 24 hours after seeding showed a well spread and smooth endothelial monolayer, with few areas of exposed capsule surface (Figure 4D). This morphology was maintained for at least 14 days under static culture conditions.

### Growth rates of encapsulated smooth muscle cells

**Figure 5:**
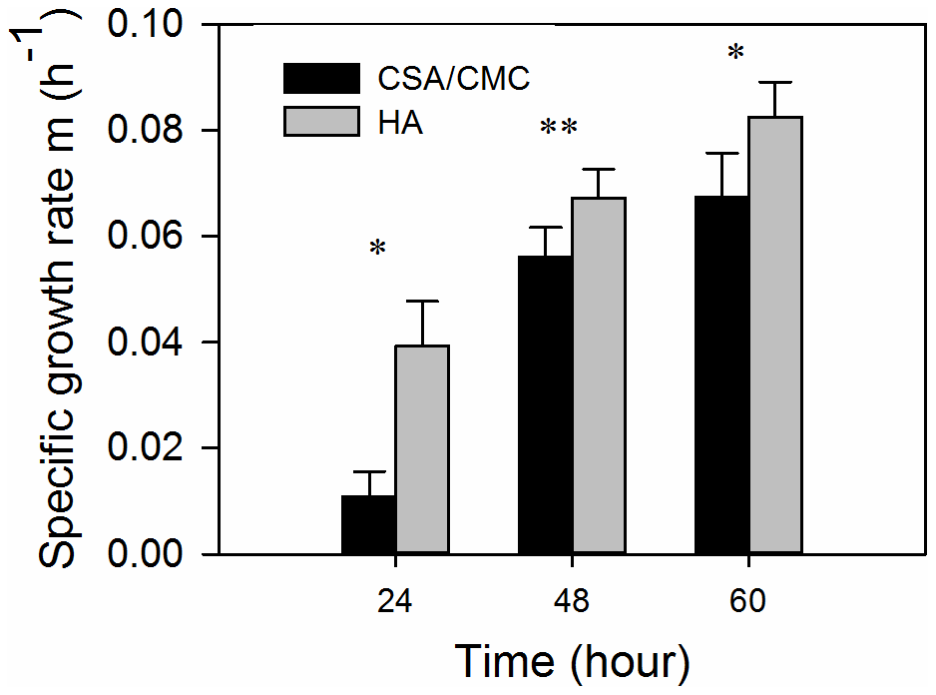
Specific growth rates of aortic smooth muscle cells in HA and CSA/CMC capsules. Specific growth rates were calculated using DNA measurements. Error bars represent standard deviations of 3–5 independent measurements. Significant differences are denoted by single or double asterix (*  =  p<0.05; **  =  p<0.10).

Sheep aortic smooth muscle cells were encapsulated for purposes of evaluating the performance of a normal parenchymal cell type. Use of these cells also allowed indirect evaluation of their interaction with endothelial cells in a subsequent study. SMC specific growth rate data showed that the cells proliferated significantly better in HA than in CSA/CMC capsules (p<0.05) during the first 36 hours of culture (Figure 5). However, differences between the formulations were less pronounced after 36 hours (p<0.10). The difference in cell proliferation might be attributable to hyaluronan-specific signaling through CD44 cell surface receptors [44]. Alternatively, CSA, a sulfated GAG, may have bound and partially sequestered growth factors necessary for SMC growth. In addition, the HA capsules appeared to support slightly better cell attachment to the internal surface than CSA/CMC, thereby promoting formation of several small aggregates rather than the single large spheroid typically seen in CSA/CMC capsules. Smaller aggregates are less likely to be adversely affected by diffusion limitations and may thus exhibit higher growth rates in the early stages than larger aggregates.

### Capsule membrane permeability

**Figure 6:**
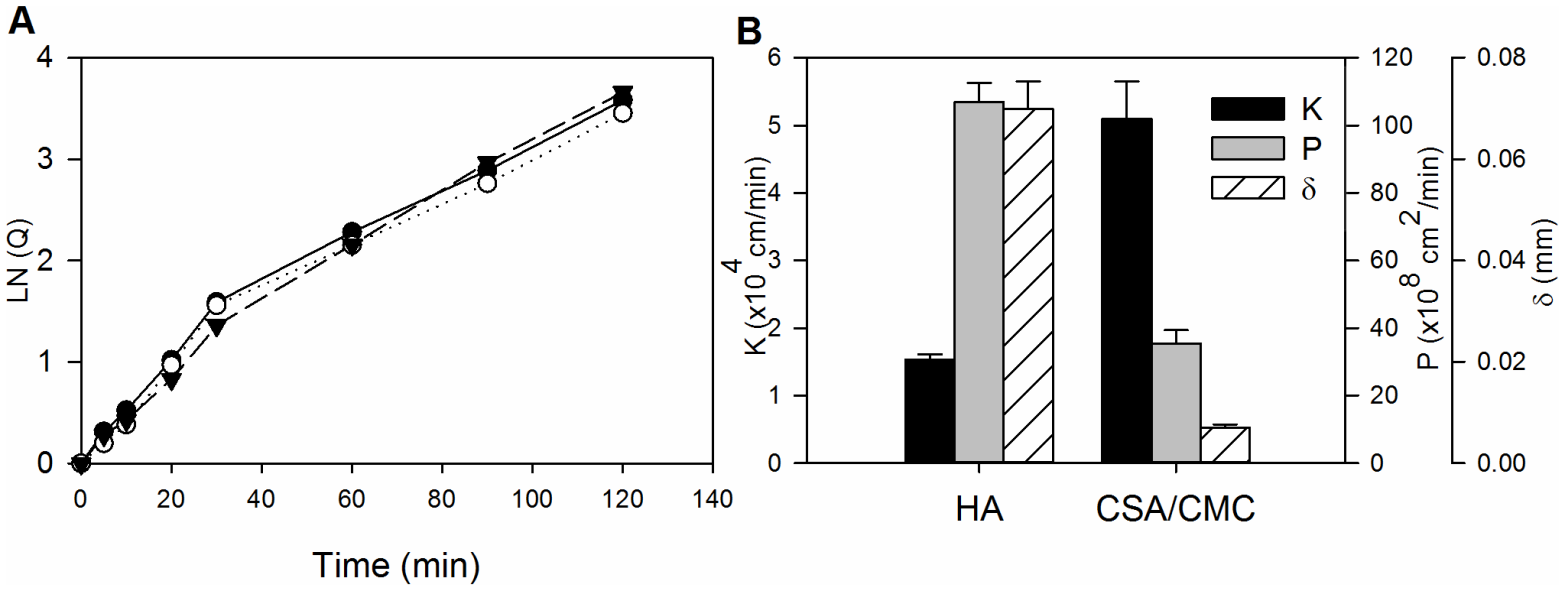
Albumin permeability measurements and mass transfer characteristics of HA and CSA/CMC capsules. (A) Representative plots of the concentration factor ln(Q) vs. time for three replicate runs with the CSA/CMC capsule formulation. (B) Plots of permeability coefficient (P), overall mass transfer coefficient (K) and wall thickness (δ) for the HA and CSA/CMC capsule formulations. Error bars represent the standard deviation of three replicate measurements.

Results of the diffusion studies on encapsulated BSA are shown in Figure 6. A typical plot of the dimensionless concentration factor ln (Q) vs. time, for three replicates runs of the CSA/CMC capsule formulation is shown in Figure 6A. The higher slope of the curve observed at early time points, is likely due to the rapid desorption of weakly bound albumin from the capsule wall. For our diffusion calculations, only the slope of the later, linear portion of the curve was used. Figure 6B compares the values of the overall mass-transfer coefficient (K), permeability coefficient (P), and wall thickness (δ) for the two most stable capsule formulations (HA and CSA/CMC). As expected, the HA capsules exhibited ∼3 fold higher permeability than CSA/CMC capsules due to the higher molecular mass of HA and its expected formation of a looser polyelectrolyte complex network with chitosan. However, the overall mass transfer coefficient which correlates directly with the overall rate of BSA diffusion from capsules was higher in the CSA/CMC capsules, mainly due to their thinner walls. Significant post-formation swelling was also observed in CSA/CMC capsules, which nearly doubled their initial diameter. No such swelling was observed with HA capsules. Overall, the results indicate that both capsule types are permeable to globular proteins of moderate size, and suggest that the permeability of the capsule wall might be further tunable via the molecular weight of the capsule materials.

### Tuning the interior capsule microenvironment

**Figure 7:**
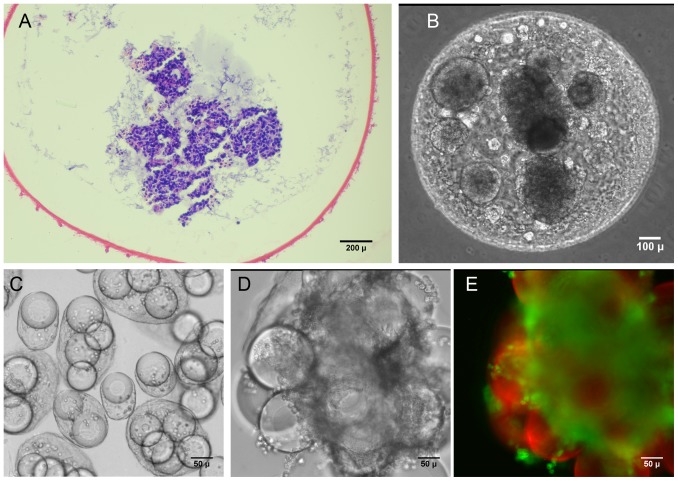
Tuning the inner capsule microenvironment with a collagen gel matrix and microcarriers. (A–B) HTBs in CSA/CMC capsules with a collagen type-I gel after one week of static culture. (A) H&E histology. (B) Phase contrast image. (C–E) SMCs co-encapsulated with gelatin coated dextran (Cytodex-3) microcarriers in HA capsules. (C) 60 min after encapsulation. (D) Day 14 of culture. (E) Calcein-AM stained fluorescence images on day 14 (green  =  live cells, red  =  microcarriers).

The hollow nature of the GAG-based microcapsules allowed us to tune the inner microenvironment by co-encapsulating additional materials. We investigated the effect of incorporating a collagen gel matrix and the inclusion of microcarriers by co-encapsulating them in microcapsules along with cells. When a collagen matrix was included along with the HTBs, the aggregates formed were smaller, more numerous, loosely organized and distributed inside the CSA/CMC capsules (Figure 7(A, B)). The collagen matrix did not appear to affect the invasiveness of the HTBs, and overall capsule integrity remained unaltered. The looser organization of HTBs in the presence of collagen was likely due to integrin-mediated cell-matrix adhesion which competed with cadherin-mediated cell-cell adhesion. This modification may be particularly useful for reducing the effective sizes of cell aggregates in larger capsules, and thereby reducing intra-aggregate diffusion limitations.

To further modulate the intracapsule environment, we investigated the effect of gelatin coated dextran microcarriers (Cytodex-3, Sigma) encapsulated in HA and CSA/CMC capsules, on aortic smooth muscle cell proliferation and viability. The inclusion of microcarriers in the capsules reduced the formation of large aggregates and dispersed the cells more evenly across the capsule. Interestingly, the inclusion of the microcarriers in the polyanionic solution resulted in greatly reduced swelling and smaller sized capsules (Figure 7C) compared to capsules formed without microcarriers. Calcein AM and Ethidium Homodimer fluorescence imaging after 12–14 days of encapsulated culture showed that encapsulation of microcarriers along with the cells promoted microcarrier adhesion and proliferation of SMCs as shown in Figure 7(C, D, E). Similar adhesion and growth results were not observed in capsules without microcarriers or with microcarriers made of dextran alone (Cytodex-1, Sigma).

### Cell-contractable capsules for higher density cultures

**Figure 8:**
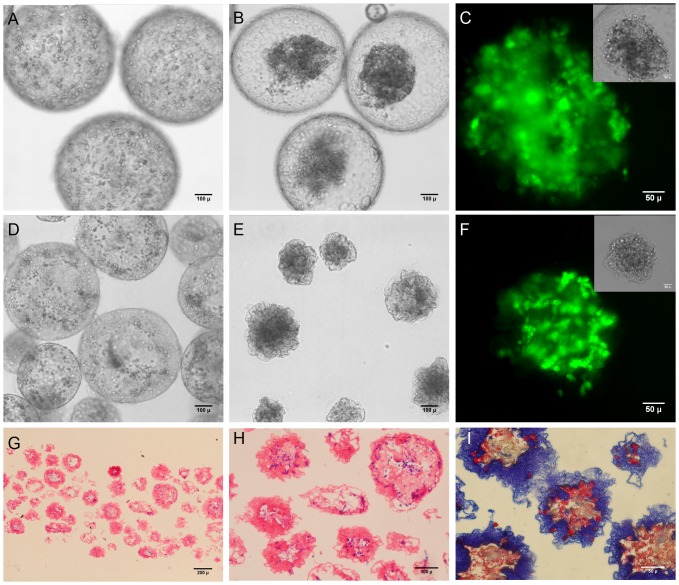
Vascular smooth muscle cells in collagen-containing capsules. (A–C) SMCs in CSA/CMC capsules with a 1 mg/ml collagen gel. (A) 60 min after encapsulation, SMCs are well dispersed in the internal collagen matrix. (B) After 24 hours of culture, the cells had contracted the internal collagen gel and formed a dense cell-matrix mass. (C) Calcein-AM fluorescence of contracted cell mass. Inset shows phase contrast image. (D–F) SMC encapsulated in HA capsules with 1 mg/ml collagen-I gel. (D) 60 min after encapsulation, cells are well dispersed in the internal collagen matrix. (E) After 24 hours of culture, the cells contracted the internal collagen gel, simultaneously collapsing the entire capsule structure to form a denser module with a convoluted surface membrane. (F) Calcein-AM fluorescence of contracted cell mass. Inset shows phase contrast image. (G–I) Histology of contracted capsules. H&E (G,H) staining showing compacted capsule structure with minimal void volume. (I) Masson's Trichrome staining of contracted capsule, showing the distribution of collagen (blue) within the structure.

**Figure 9:**
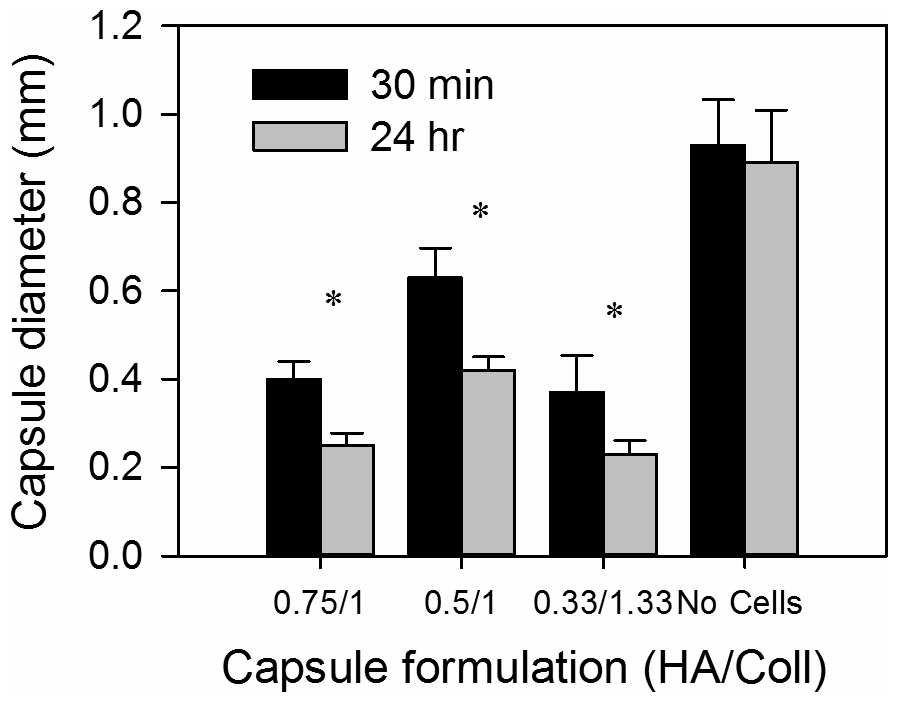
Effects of HA and collagen concentrations on cell-mediated capsule contraction. Maximal cell-mediated contraction was seen in the formulation with a final concentration of 0.33 wt% HA/1.33 mg/ml collagen-I. Capsules without cells exhibited an insignificant reduction in capsule diameter. Error bars represent standard deviation from at least 10 capsule measurements. Asterix denote statistically significant differences (p<0.05).

Some cell types may not grow well in a GAG-only ECM environment and hence attaining high cell densities could be a challenge. To address this, we developed a capsule formulation that promoted the cell-mediated contraction of capsules and the rapid elimination of excess capsule volume. We investigated the ability of collagen to promote contraction of the capsules by encapsulating smooth muscle cells with varying volume ratios of collagen gelling solution to GAG solution. Initially when collagen was encapsulated along with HTBs in CSA/CMC capsules at a final concentration of 1.5 mg/ml, the growing cells formed aggregates which were loosely packed as discussed earlier (Figure 7 A–B). When collagen of the same concentration was encapsulated along with SMCs at a similar cell density in CSA/CMC capsules, the cells were initially uniformly dispersed within the collagen gel inside the capsules. However, within 24 hrs the SMCs contracted the collagen matrix and formed a denser mass of cells and matrix as shown in Figure 8 (A, B, C). Even though the internal matrix was contracted, the walls of the CSA/CMC capsules were unyielding and retained their spherical shape (Figure 8B). Similar experiments were conducted with SMCs in HA capsules using various concentrations of HA and collagen-I to examine the collagen-mediated contraction effect. HA capsules containing SMCs suspended in a collagen gel were found to support cell-mediated contraction and crumpling of the entire capsule wall simultaneously with contraction of the interior gel (Figure 8 D, E, F). Sectioning and staining (H&E and Trichrome) revealed a structure with cells embedded within a dense collagen matrix inside the capsule and also large amount of collagen integrated into the capsule walls (Figure 8(G, H, I)). Maximal contraction with ∼75% reduction in volume (∼37% reduction in diameter) was achieved using a polyanion solution containing 0.33 wt% HA and 1.3–1.4 mg/ml of collagen-I (Figure 9). The capsule contraction was confirmed to be cell mediated based on the insignificant reduction in capsule diameter of cell-free capsules (Figure 9). This collapsible capsule formulation may be a useful tool for preparing reduced diameter capsules with higher density cell content, for use with cells that do not proliferate well in-vitro. The technology may also be utilized to improve diffusional transport performance in encapsulated culture by reducing the excess capsule volume and effectively increasing the surface to volume ratio.

### Cocultures of smooth muscle and endothelial cells

**Figure 10:**
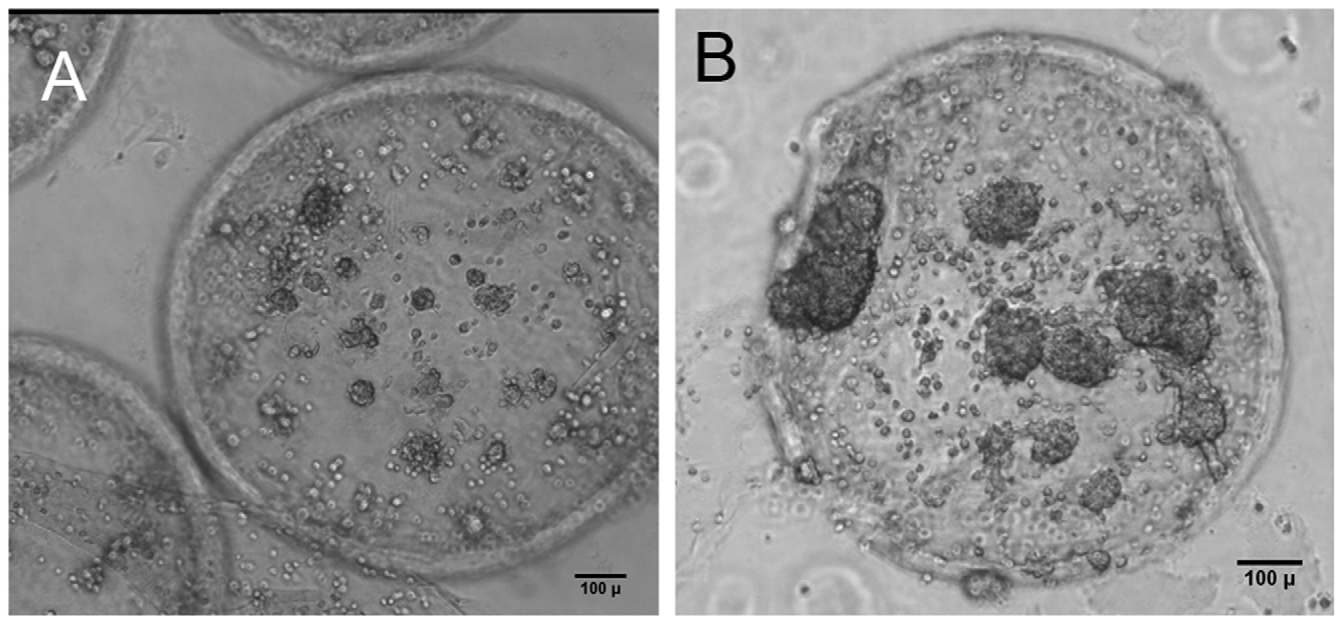
Cell growth in encapsulated cocultures of SMC and AEC. Cocultures of encapsulated SMCs with AECs on the external surfaces of HA/Collagen capsules exhibited increased SMC proliferation compared to encapsulated SMC monocultures. (A) Encapsulated SMCs only, day 7. (B) Encapsulated SMC with AECs, day 7.

Many studies have demonstrated functional relationships between endothelium and adjacent cell types [45]–[49], [50], [51]. Designing tissue assembly approaches that allow critical paracrine interactions is mandatory for achieving in vivo-like performance of engineered tissues. Towards this end, we sought to characterize the growth of encapsulated smooth muscle cells with and without capsule surface-seeded endothelial cells in HA capsules. Phase contrast imaging showed that encapsulated SMCs co-cultured with AECs on the external capsules surfaces exhibited greater proliferation than SMC-only capsules, as indicated by the larger SMC aggregates seen in Figure 10B. This result suggests that the HA chitosan membrane possessed enough permeability to allow stimulatory paracrine signaling between AECs and SMCs.

### Assembly and perfusion bioreactor culture of capsule modules

**Figure 11:**
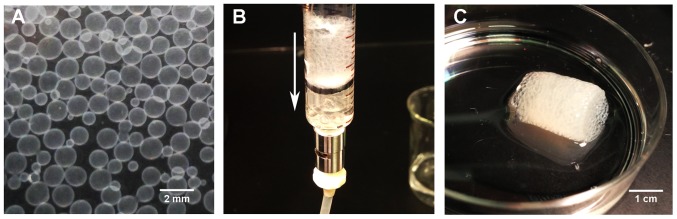
Modular assembly of GAG based microcapsules by fusion. Modular constructs were fabricated by perfusing packed capsules in a chamber of desired dimensions with diluted polymer solutions. This method yielded self-supporting constructs with uniform porosity. (A) Individual capsules in buffer solution before fusion. (B) Capsules being perfused with polymer solution in a perfusion chamber. Arrow indicates direction of flow. (C) Fused construct after removal from perfusion chamber.

**Figure 12:**
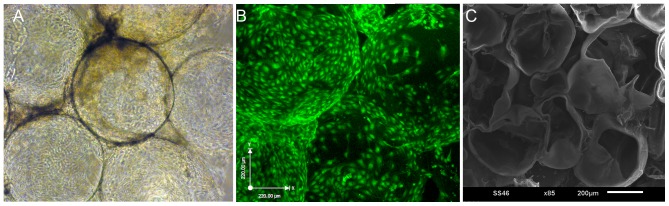
Endothelialized, interconnected channels in a fused modular construct. (A) Phase contrast image of CSA/CMC capsules, seeded externally with HUVECs and fused 48 hours after seeding. (B) Combined confocal image stack of the modular construct shown in A with HUVECS visualized via CellTracker Green staining. (C) SEM image of an axially sectioned, modular construct assembled from fused empty capsules showing interconnected channels.

Assembly of capsules into three-dimensional modular constructs is fabrication critical step in our modular tissue engineering approach to generating vascularized tissue. We investigated various methods for assembling larger 3D constructs from pre-cultured individual capsules. The most successful method involved reloading cultured capsules with a polyanion, followed by perfusion with a diluted polycation solution. Outward diffusion of reloaded GAG during the polycation perfusion step deposited a polyelectrolyte complex that effectively fused capsules together around points of contact. This method yielded self-supporting structures with interconnecting, perfusable spaces as shown in Figures 11 and 12.

Capsules that were surface-seeded with HUVECs and subsequently fused showed well endothelialized, interconnected channels as seen in phase contrast (Figure 12A) and confocal images (Figure 12B). SEM imaging of an axial section through a fused construct shows the interconnected channels more clearly (Figure 12C).

**Figure 13:**
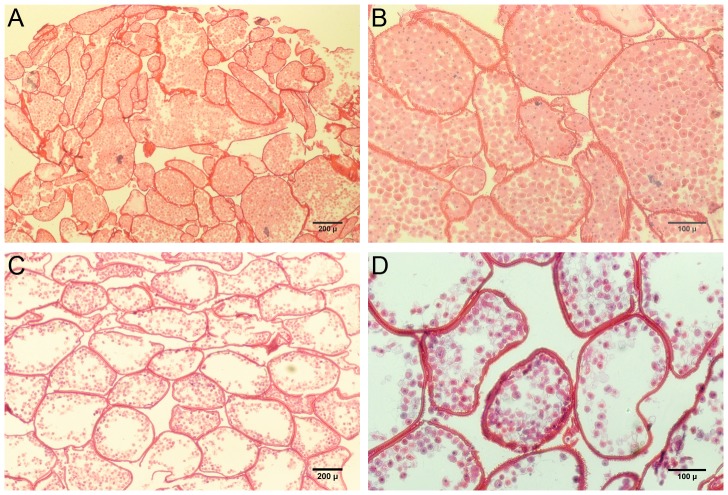
H&E staining of modular constructs based on hepatocytes in HA/collagen capsules. (A, B). Fused construct with reduced fluid volume and porosity due to centrifugation of capsules during the fusion process. (C, D) Construct formed by fusion of capsules settled under unit gravity, resulting in significantly greater fuid volume inside capsules and larger intercapsular spaces suitable for perfusion culture.

In a modified procedure, fusion-based assembly of high cell density capsules was explored by encapsulating primary rat hepatocytes in HA-collagen capsules at a density of 10×10^6^ cells/ml of the HA/collagen solution, followed by heparin reloading, and centrifugation for 10 seconds at 50G to expel excess intracapsular liquid. H&E stained sections of the resulting construct showed a dense cell mass (∼5×10^7^ cells/cm^3^, estimated via image analysis) with reduced, but still significant intercapsule spaces (Figure 13 A–B). Encapsulated hepatocyte constructs assembled without centrifugation showed a much less dense cellular construct (estimated at 9×10^6^ cells/cm^3^ via image analysis) with more and larger intercapsule spaces (Figure 13 C–D). These results demonstrate that additional physical processing methods can be used to further adjust the effective cell density and perfusable void space within these modular constructs.

**Figure 14:**
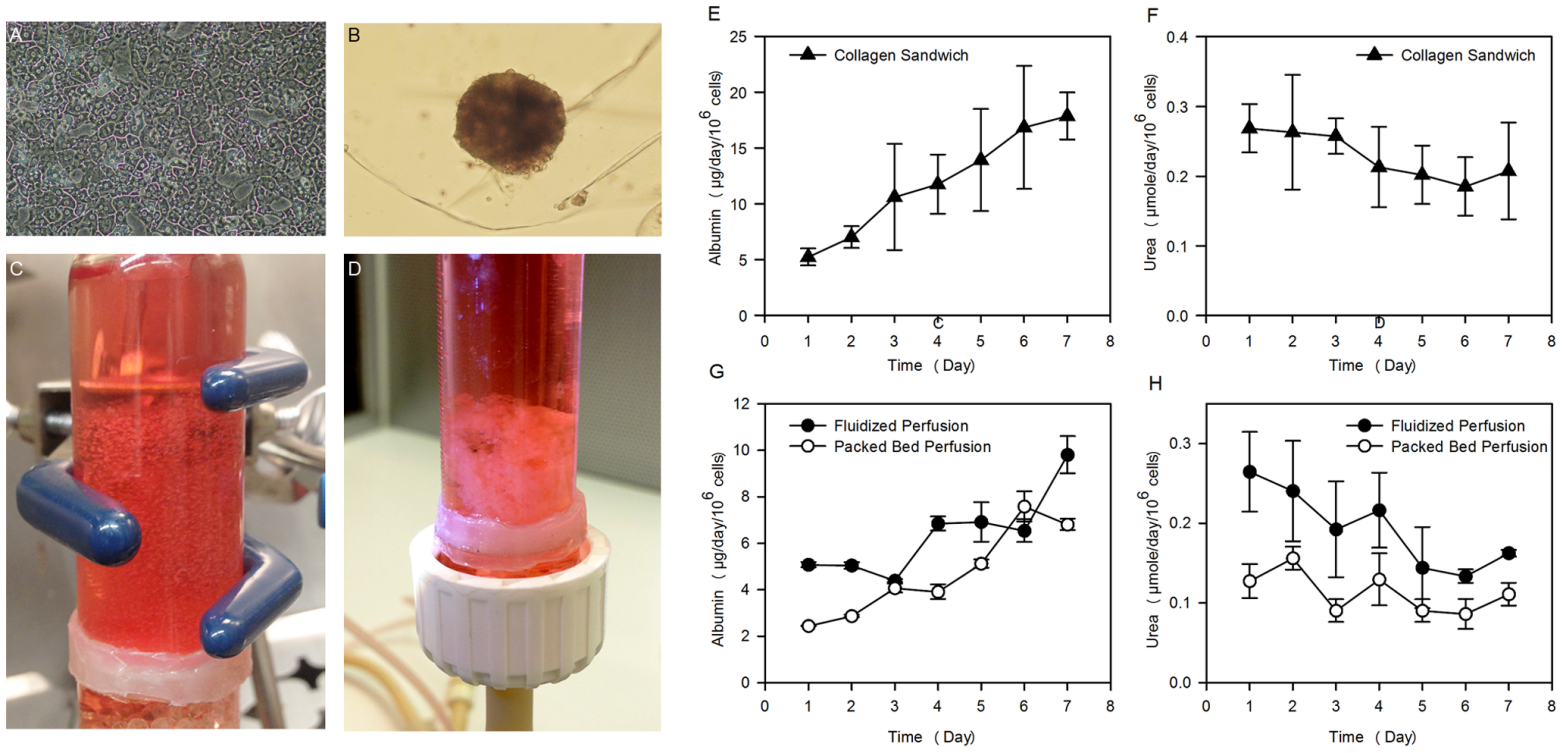
Albumin and urea synthesis rates of hepatocytes in encapsulated perfusion cultures. Primary rat hepatocytes were encapsulated in CSA/CMC capsules with a with 1 mg/ml collagen gel, at a density of 2×10^7^ cells/ml of CSA/CMC/collagen solution. (A) Control collagen sandwich dish culture. (B) Encapsulated hepatocytes aggregated into spheroids during culture as either (C) individual capsules in a fluidized bed bioreactor, or as a (D) fused modular construct in a packed bed bioreactor. (E–H) Albumin and urea synthesis rates by the hepatocytes in the three culture conditions. (E,F) Control collagen sandwich cultures. (G, H) Perfusion cultures. Error bars denote standard deviations from 3 replicate measurements.

The metabolic performance of encapsulated primary rat hepatocytes maintained in perfusion culture conditions (Figure 14 C–D) was evaluated by measuring urea and albumin synthesis rates and comparing to rates of identical cells in standard collagen sandwich dish cultures (Figure 14A). Perfusion cultures maintained the functionality of encapsulated hepatocytes and healthy spheroids were seen in most capsules (Figure 14B). Albumin and urea synthesis rates in both types of perfusion cultures (Figure 14 G,H) approached those of the collagen sandwich cultures (Figure 14 E–F).

## Discussion

Modularity is a phenomenon widely observed in nature, which enables biological systems to achieve precise control over organization and function in very compact spaces. The modularity of the kidney and its component nephrons are excellent examples of this concept. Adopting a similar approach in engineered organs has a number of advantages. The scalability of the modular strategy enables rapid fabrication of tissue constructs with greater control over their architecture. The major design challenges of a modular tissue construct include: limiting mass transfer distances, achieving high, tissue-like cell densities, and the ability to form interconnected, vascularizable channels. The GAG-based microcapsules described here allow efficient mass transfer, which is evident from the tissue-density cultures that were maintained for up to 45 days under static culture conditions. The diameter of the capsules can be easily controlled between 0.3 and 2.0 mm, and smaller diameters are achievable using more sophisticated droplet formation methods such as microfluidics. Capsule diameter imposes a natural upper limit on the maximum diffusion distances. The capsule system, in particular the hyaluronan-based capsules, supports direct encapsulation of cells at high, in vivo-like densities. In addition, the cell-contractable capsule formulations provide an additional mechanism for modulating cell density within either the capsules or the fused construct. Under random packing conditions, capsule fusion produced 3D structures with significant void space available for direct perfusion, accessory cell culture, or vascularization. The dimensions and architecture of the intercapsular voids can also be modulated by incorporating additional biomaterial components into fused capsule structures. Such accessory components include fibers, beads, films, tubes, etc. made from chitosan, chitosan-GAG complexes, or other degradable materials. The materials used in our modules are fully degradable, and previous implantation results with similar materials indicate that in contrast to pure chitosan [52], chitosan-GAG complexes [53] degrade rapidly in vivo and stimulate rapid and extensive neovascularization due to GAG-mediated effects [54]–[58]. The high density trophoblast cultures were primarily intended to demonstrate the potential of the microcapsules with a highly proliferative human cell type. However, these cultures also provided direct evidence of both the degradability of the GAG-chitosan materials, and the ability of cells to invade the capsule wall. The trophoblast cell line maintains some characteristics of human trophoblasts, in particular the ability to tolerate hypoxic conditions and to invade tissue rapidly. Both characteristics are presumably related to its original, placenta-formation function [59] and may be mediated by focal expression of MMPs, GAG lyases or other matrix degrading enzymes. Wall invasion and cell escape in these trophoblast cultures was evident after week 2 of culture and was clearly captured in histological sections (Figure 3C). This phenomenon strongly suggests that implanted capsules would present only a temporary barrier to integration of encapsulated cells with adjacent tissues. Coupled with the known pro-angiogenic effects of GAG-based materials [60]–[62], these results further suggest that rapid vascularization is a likely outcome after transplantation of capsule-based constructs

Beyond modular assembly, the ability to incorporate clinically significant cell numbers into an implantable construct of feasible size is an additional challenge. We have shown that cells and matrix can be efficiently packed inside capsules of a non-diffusion limited size (Figures 8, 13). Our liver organoid prototype had a cell density of 50×10^6^ cells/cm^3^ (Figure 13A). This is 40-60% of the hepatocyte cell density of liver tissue. From a practical standpoint, the cell densities achieved in our systems are adequate for liver tissue engineering, as it has been demonstrated that with good blood chemistry, ∼10% of total liver mass can support survival in rats [63], [64] and humans [65]. It should be noted that maintaining high cell densities inside capsules presents particular diffusion challenges in the case of highly metabolic cells such as primary hepatocytes. Thus, we have also shown that the cell density can be scaled down to compensate for such high metabolic requirements (Figure 13 C–D). In principle, diffusion challenges can be minimized by limiting the maximum capsule diameter to ensure an adequate supply of nutrients and oxygen to all regions of the cell mass. Diffusion inside capsules can be further modulated by controlling the extent of cell distribution and aggregation. In particular, co-encapsulating hydrogel components (e.g. collagen gels) or microcarriers provides a mechanism for tuning the interior microenvironment as well as the architecture of the cell mass. Such hydrogel materials can benignly interfere or directly compete with large scale cell aggregation, and thus serve to promote formation of multiple smaller or looser cell aggregates.

The encapsulation method also allows incorporation of microcarriers of various biomaterials. As with hydrogels, these microcarriers can produce additional adhesion ligand signaling, organizational barriers or mechanical enhancement. Our results show that gelatin coated dextran microcarriers significantly enhanced the growth and viability of encapsulated smooth muscle cells. These and other cell-adhesive microparticles can also be used to alter the physical properties of the fused capsule construct. It should also be noted that inclusion of microcarriers resulted in capsules with reduced osmotic swelling and substantially reduced internal volumes. This was particularly noteworthy in the case of CSA/CMC capsules which swelled more than HA capsules. We postulate that the increased swelling in the CSA/CMC system was due to higher interior osmotic pressures resulting from combined effects of a higher mass concentration, and lower molecular mass of the interior polymer solution compared to HA capsules. Inclusion of a high volume fraction of microcarriers within a capsule-forming CSA/CMC droplet reduced both the volume of CSA/CMC solution inside the capsule and also the residual concentration of this solution after capsule membrane formation. This lower final concentration (caused by incorporation of polymer into the capsule membrane) produced a lower osmotic pressure and resulted in contraction of the capsule membrane around the cell+microcarrier mass. In general, the inclusion of microparticles provides a wide range of options for tuning the cellular organization and overall mechanical properties of modular constructs.

Fused capsule modules yielded 3D constructs with inter-capsular spaces that are perfusable in vitro and potentially vascularizable in vivo. The urea and albumin synthesis rates of the perfused cultures indicate that mass transfer rates were sufficient to maintain the encapsulated hepatocytes in our modular constructs. In addition, the interconnected endothelilized channels may provide a foundation for a vascular network and thereby accelerate the process of neovascularization by anastomosing with the host vasculature post-implantation. At the very least, intercapsular endothelial cells are likely to participate in vessel formation between fused capsules. However, the kinetics of this process, and the relative degrees of transplanted vs. host cell organization in the final structure remain to be characterized through animal studies.

Our results also suggest that the capsule membrane can facilitate paracrine signaling as seen by the increase in SMC proliferation during coculture with endothelial cells. This suggests that various other interacting cell types can be cultured in this modular system with a degree of material-based control over cell organization while still allowing substantial paracrine signaling. Several coculture systems have previously been shown to improve morphology and function of engineered tissues including liver [66]–[70], bone [71], [72], [73] and cartilage [74]–[76]. Our results suggest that similar trophic effects can be achieved with ease in capsule-based modular scaffolds, with added the added benefit of control over cell arrangement and distribution.

Unlike traditional scaffolds, porosity can be either maintained evenly throughout the modular capsule scaffolds or different layers with different capsule sizes and hence different porosity can be easily implemented. GAG-chitosan surfaces can support cell adhesion and proliferation, partly due to GAG-mediated binding of matrix proteins and growth factors [53], [77]. External cell adhesion can further be enhanced by directly incorporating cell-adhesive proteins such as collagen into the capsule wall by either blending with the polycationic solution or direct application to external capsule surfaces.

In conclusion, we have demonstrated the formation and use of GAG-based microcapsules to generate a variety of tunable, intracapsular microenvironments. These capsules have been shown suitable for fabrication of porous, 3D constructs that have the potential to mimic native tissue architecture with high cell densities, vascular and parenchymal cell types, and perfusable, endothelium-lined channels. This capsule-based modular tissue assembly approach is a promising strategy that provides a wide range of options for the efficient assembly of three-dimensional, engineered tissues.
